# Closing Editorial: Conversations in healthcare education

**DOI:** 10.15694/mep.2019.000149.1

**Published:** 2019-07-08

**Authors:** Stuart Marshall, Debra Kiegaldie

**Affiliations:** 1 Monash University; 2 Holmesglen Institute

**Keywords:** Clinical Skills, Clinical Conversations, Communication, Feedback, Debriefing, Teamwork, Collaboration

## Abstract

This article was migrated. The article was marked as recommended.

The theme coincided with the 8 ^th^International Clinical Skills Conference held in Prato Italy, May 2019. The conference focused on putting the spotlight on conversations in healthcare and we were highly successful in achieving this aim by generating much discussion, debate and dialogue amongst our conference delegates. The conference is known for its informality, friendliness and ability to bring people together as a community of practice and this year was no exception. The conversations on twitter alone was unprecedented with over three million impressions over the period of the conference. This is a truly remarkable number given that the conference is limited to only 250 delegates because of the size of the venue.

Whilst the conference did not generate as many paper submissions to MedEdPublish as we would have liked, the five papers submitted were noteworthy and included practical tips to use for teaching intrinsic skills to physicians ( LaFleur *et al*., 2019), implementing a collaborative social media platform in obstetrics and gynaecology ( O’sullivan, Janssens and Warhurst, 2019), and developing consultation skills for medical students ( Andersson *et al*., 2019).

Nevertheless, we believe this is the first experiment in true 360-degree scientific peer review. A system where manuscripts published on the journal’s website before a conference can be explained in conference presentations, questions asked, discussed on social media and finally reviews published alongside the paper on the website. Such a model must surely be beneficial for disseminating new research and ensuring it is of the highest standard.

Conversations in healthcare today continue to be of great importance to the future of clinical education and for developing health professionals with the skills needed for person-centred care and we look forward to continuing this work into the future.

## Conference keynote conversations

Our keynote speakers stimulated much conversation throughout the conference and on twitter when presenting their work about clinical conversations.

Liz Crowe opened the proceedings and discussed the cost of incivility on humans in relation to error, safety, retention and wellbeing. She highlighted how our leaders and teams often do not know what to do about incivility and the impact this has on individuals. She talked about learning to speak the language of responsibility and the need for robust conversations; and the imperative to ensure that destructive conflict has more open communication.

“Conflict has the power to transform. Conflict can only be constructive rather than destructive when conversations occur from a space of shared vulnerability, where language is spoken with responsibility and ownership and when people are less interested in justification and more interested in curiosity and understanding.” ( Crowe, 2019)

Tanja Manser’s inspiring presentation on ‘team talk’ emphasised communication among healthcare professionals to ensure safe and effective team communication and patient care. She described communication behaviours and meta-communicative strategies including implicit coordination between team members that can occur after extensive training together ( Manser, 2019)

Jimmie Leppink challenged the delegates to consider how to measure the process of learning and think of the longitudinal measurement of students. By using control charts, network mapping and other statistical techniques, students in difficulty could be identified and remediated earlier ( Leppink, 2019).

Another perspective was presented by Walter Eppich in relation to ‘learning through talk‘. His work and body of research has examined ‘talk’ as a joint social activity between conversation partners and as a way of learning for practice. Through simulation and workplace learning he addressed the question of: How does talk contribute to learning in clinical education? He explored the structured talk of feedback and debriefing in healthcare simulation that attends to both the process and the content of learning conversations. He concluded that:

“Health professions educators should steer the talk of practice in two ways: (a) through formal and informal means, such as structured feedback and debriefings that attend to relevant process and content, and strategies that foster relationships and supportive learning environments, and (b) through simulations designed to sensitise clinicians to the affordances of future workplace talk.” ( Eppich, 2019)

Finally, Vicki Le Blanc provided the delegates with an insightful presentation on rethinking the role of emotions in learning and clinical skills. She spoke about how emotions can have a significant impact on how we perceive the world around us, what we pay attention to, what we remember, as well as our judgements and decision making. She highlighted the importance for clinicians to recognise how emotional states affect our ability to interpret information, make decisions and remember critical information.

“This greater understanding will, in turn, shape how we teach, provide feedback and coaching, as we as how we assess our learners.” (LeBlanc, 2019)

## Conference conversations

In keeping with the conference theme, a small number of delegate abstracts and presentations explored the nature and process of clinical conversations. Mcallister, *et al.* (2019) presented work on preparing students for intentional conversations with older adults by using narrative skills to increase networks of communication with older people. The authors described a concept called ‘Narrative Competence’ - that is the ability to deeply listen to a person’s story and then to communicate that story back so that it becomes life-enhancing ( Corbaly and Grant, 2015). It was presented as a novel solution to a very difficult problem. The authors found that Narrative Competence developed nursing student’s intentional conversational skills, emphatic engagement, communication, cultural competence, collaborative goal setting and fostered a sense of community.

Purcell described an Accelerate Communication Excellence (ACE) programme for health students designed to address issues of poor communication competence ( Purcell, 2019). ACE includes a web-based diagnostic program on professional communication and a 4-day immersive program. Outcomes of the evaluation revealed diagnostic accuracy for students with poor professional communication competence and development of listening, reading, writing, reasoning and speaking skills in novice students in the immersive programme.

One additional innovative presentation was a project conducted by McNeill *et al.* (2019) that used Lego® as a fun way for midwifery and medical students to get to know each other, initiate conversations and bond as a team prior to working in simulations together. They found that the activity enabled open discussion, laughter and team building.

## MedEdPublish conversations

Six manuscripts were accepted from those submitted. The papers published comprised four research articles, a new education method and a practical tips paper. Authors selected a variety of keywords to portray their papers association with our theme including: non-medical expert roles; clinical skills; CanMEDS intrinsic roles; competency-based medical education, social media, online forum, consultation skills; real patients; video-recordings; Cambridge-Calgary Global Consultation Rating Scale; patient centeredness and empathy.

The most relevant paper that contributed to our theme was the practical tips and/or guidelines paper written by Lafleur, Gagne, Paquin and Michaud-Couture from the Universite Laval ( Lafleur, Gagne, Paquin *et al*., 2019). They provided a matrix of high impact clinical studies exemplifying intrinsic roles of physicians. By intrinsic roles the authors referred to the CanMEDS framework for non-medical roles i.e. communicator, collaborator, manager, scholar, professional and health advocate. In their article, the authors provided *practical tips* on why and how to use high-impact clinical studies to enlighten supervisors and trainees about the educational and clinical importance of those skills. It was also accompanied by a slide kit for use in clinical settings and a selection of 30 examples of ‘hard’ evidence on intrinsic skills with the aim of reinforcing the fact that intrinsic roles are intertwined with the medical expert role to improve patient care.

## Take Home Messages

As editors for this edition and from reviewing the papers and abstracts submitted in association for this conference we are reminded of how diverse and important clinical conversations are in healthcare today and the need for greater research into this area. Clinical conversations are not always easy to have or to teach but when done well can make a difference to the lives of our patients, families and peers. They are the lifeblood of education, should not be taken lightly and should be firmly and explicitly embedded into educational delivery.

## Notes On Contributors

Associate Professor Marshall is the chair of the International Clinical Skills Conference, a practising anaesthetist and simulation educator and researcher.

Professor Kiegaldie is the Clinical Chair of Health Workforce and Simulation at the Holmesglen Institute.

## Declarations

Debra Kiegaldie and Stuart Marshall are guest theme editors for the “Conversations about Clinical Skills” themed issue in AMEE MedEdPublish. Stuart Marshall is the Chair of the International Clinical Skills Conference committee and International Clinical Skills Foundation (Inc.).
