# Supplementary material for: Closing Editorial: Conversations in healthcare education
Source: MedEdPublish (2016). 2019 Jul 8;8:149. [Version 1] doi: 10.15694/mep.2019.000149.1 (PMC10712459; doi:10.15694/mep.2019.000149.1)
Supplement: Peer review history

# Reviewer report: Reviewer response for version 1

**DOI:** 10.21956/mep.19872.r30040

**Published:** 2019-07-13

**Authors:** Ken Masters

**Affiliations:** 1 Sultan Qaboos University

This review has been migrated. The reviewer awarded 5 stars out of 5 This closing editorial gives a succinct and useful summary of the most pertinent issues raised in the presented papers. Most importantly, the relationship between the conference presentations and the articles published in MedEdPublish demonstrates the viability of a useful model of exposing one’s work to academic peers; a model in which the presentations and publications are not end points, and the links are not unidirectional, but that they are rather pivotal hubs in the wider communication web between researchers, practitioners and students.

Reviewer Expertise:

NA

No decision status is available

# Reviewer report: Reviewer response for version 1

**DOI:** 10.21956/mep.19872.r30039

**Published:** 2019-07-11

**Authors:** Richard Hays

**Affiliations:** 1 James Cook University

This review has been migrated. The reviewer awarded 5 stars out of 5 I would like to thank the Theme Co-editors for managing this small experiment in academic publishing, where we linked a journal theme on clinical skills education to a contemporaneous international conference that focused on conversations in clinical skills education. While the link produced fewer papers than we had hoped in the themed issues of MedEdPublish, valuable lessons were learned. Even though MedEdPublish has relatively rapid production timelines, it proved difficult for speakers and attendees to write papers in time for the thematic issue. This should not have been a surprise, because as a writer I know that it is difficult to produce high quality papers in a short time while recovering from jetlag. Although it is difficult in advance to pick the better papers, it may be preferable to invite more papers to be submitted prior to a conference, and then encourage reviews and comments after the presentations. nevertheless, the conference was excellent and many interesting conversations took place. It was interesting to see that much of the discussion about simulation was not so much about using models and technology for psychomotor skill building, and more about highly contextualised, more integrated learning of how and when to apply those skills. There was also an emphasis on simulation in teaching professionalism and in training for management and leadership positions. Having a difficult conversation may be made a little easier by having done it before in a 'safe' environment, with a simulated correspondent. Overall, the profile of MedEdPublish was raised during the conference. and more papers from the conference are planned for submission in the coming weeks. While these papers will not appear in the themed issue, they will still be accessible, and the conversations can continue.

Reviewer Expertise:

NA

No decision status is available
